# Acute gastric dilatation in a patient with severe anorexia nervosa: a case report

**DOI:** 10.1186/s13256-020-02575-7

**Published:** 2021-02-08

**Authors:** Tyler Pitre, Jasmine Mah, Jaclyn Vertes, Barna Tugwell

**Affiliations:** 1 Michael G. DeGroote School of Medicine (Waterloo Regional Campus), McMaster University, Hamilton, Canada; 2 Department of Medicine, Dalhousie University, Halifax, Canada; 3 Division of Endocrinology & Metabolism, Dalhousie University, Halifax, Canada

**Keywords:** Eating disorder, Anorexia, Acute gastric dilatation, Gastric dysmotility, Case report

## Abstract

**Background:**

Acute gastric dilatation (AGD) leading to gastric necrosis and perforation has been reported to be a rare but fatal complication in young patients with eating disorders, particularly anorexia nervosa.

**Case presentation:**

We report a case of a Canadian female patient presenting with mild abdominal pain, with a history of anorexia nervosa, the binge/purge subtype, who was found to have severe acute gastric dilatation on subsequent computed tomography imaging. Her clinical course was uncomplicated after gastric decompression. The cause of her AGD was thought to be secondary to dysmotility disorder caused by her anorexia nervosa.

**Conclusion:**

Our case report demonstrates the importance of clinical identification of AGD and subsequent diagnosis and management. Because of the urgency to rule out obstruction or perforation through consultation or additional imaging modalities, recognition and correct diagnosis of this condition is necessary for appropriate patient management. In addition, our case report adds to an underreported but important complication of anorexia nervosa.

## Background

Anorexia nervosa confers a high degree of mortality and morbidity, including gastrointestinal disturbances [1–3]. While not fully understood, gastric distention can result from erratic eating behaviors, which may lead to decreased gastric motility and delayed gastric emptying [4]. Although a rare occurrence, acute gastric dilatation (AGD), subsequent gastric necrosis and perforation has been reported to be the cause of death in young patients with eating disorders, particularly anorexia with the binge/purge subtype [4–6]. There is limited understanding of AGD in patients with anorexia nervosa; however, AGD must be promptly diagnosed due to the potential fatality associated with this clinical situation.We present a case of severe gastric distention in a patient with acute abdominal pain in the context of anorexia nervosa.

## Case presentation

A 21-year-old Canadian female, with known anorexia nervosa binge/purge subtype, and no other medical conditions, was admitted with mild abdominal pain following an episode of binge eating. The patient reported 5 days of constipation and abdominal distension. She was able to pass flatus but could not vomit. There was no other relevant family or social history.

On initial physical examination, her vitals were stable. She appeared severely underweight with a body mass index less than 18. She did not have signs of purging such as Russell’s knuckles. On abdominal examination, she had a distended abdomen, with no peritoneal signs. She had no signs of chronic liver disease. Cardiopulmonary examination was unremarkable.

**Fig. 1:**
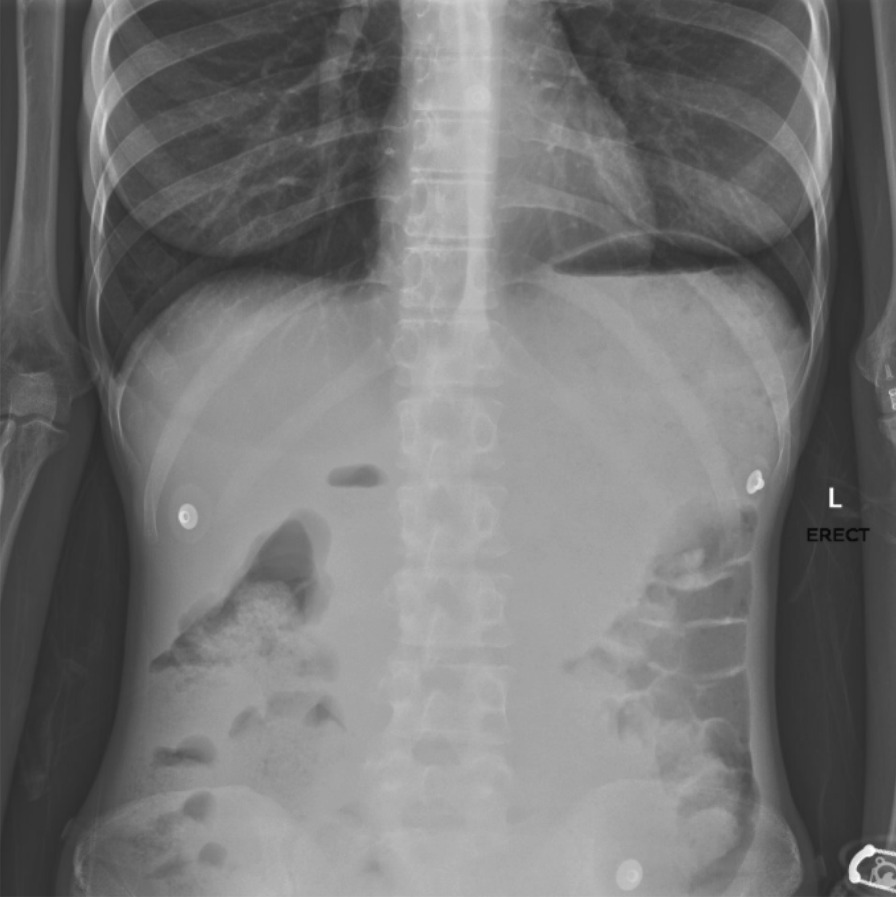
Abdominal x-ray

Abdominal x-ray revealed a large soft tissue density displacing the transverse colon inferiorly, seen in Fig. 1. There was no pneumoperitoneum and no evidence of bowel obstruction. Computed tomography (CT) of the abdomen and pelvis revealed a massively distended stomach, measuring 17 × 18 × 24 cm, approximately 4 l. There were round radiodense bodies within the stomach, which were later found to be undigested medication. There was progression of dilation into the proximal duodenum with an abrupt taper at the 3rd part of the duodenum, which was compressed as it crossed over the aorta, with collapse of distal loops of small bowel.

**Fig. 2:**
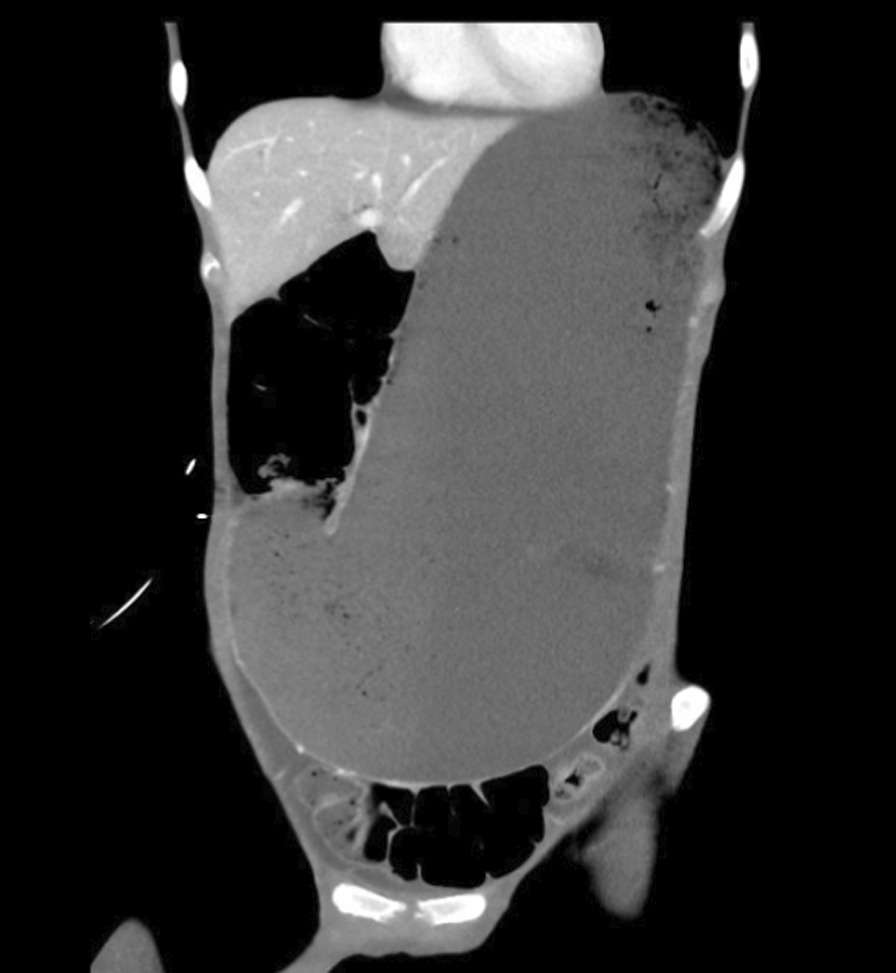
Computed tomography coronal view

**Fig. 3:**
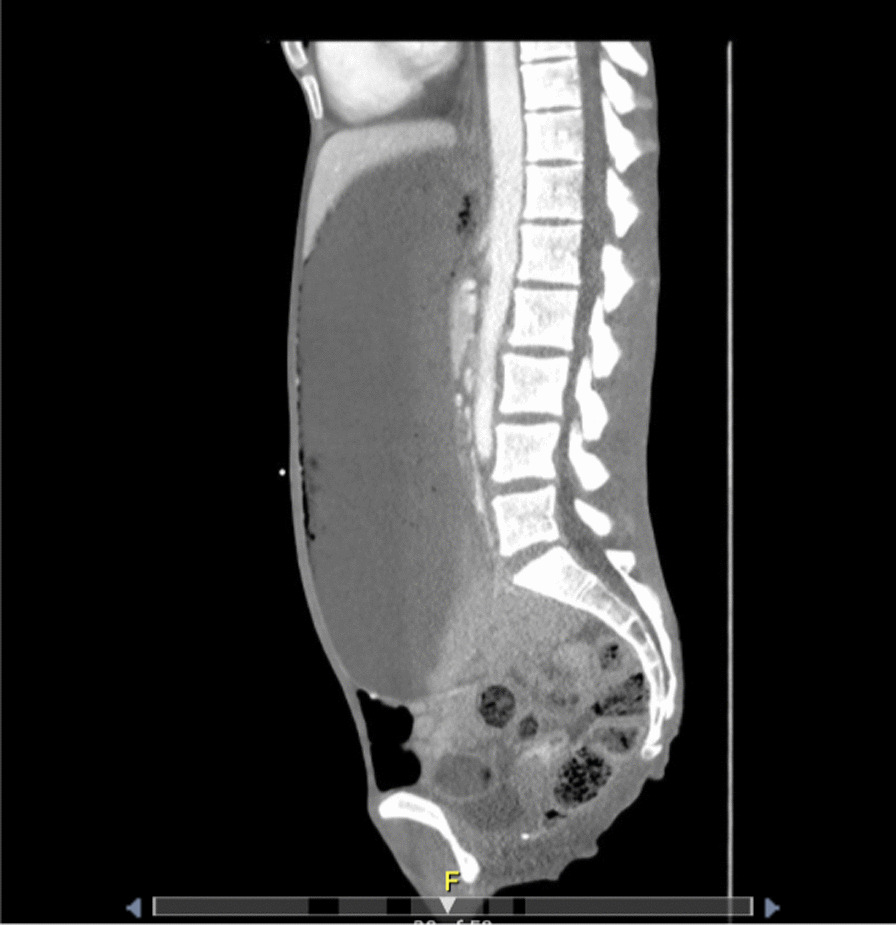
Computed tomography sagittal view

**Fig. 4:**
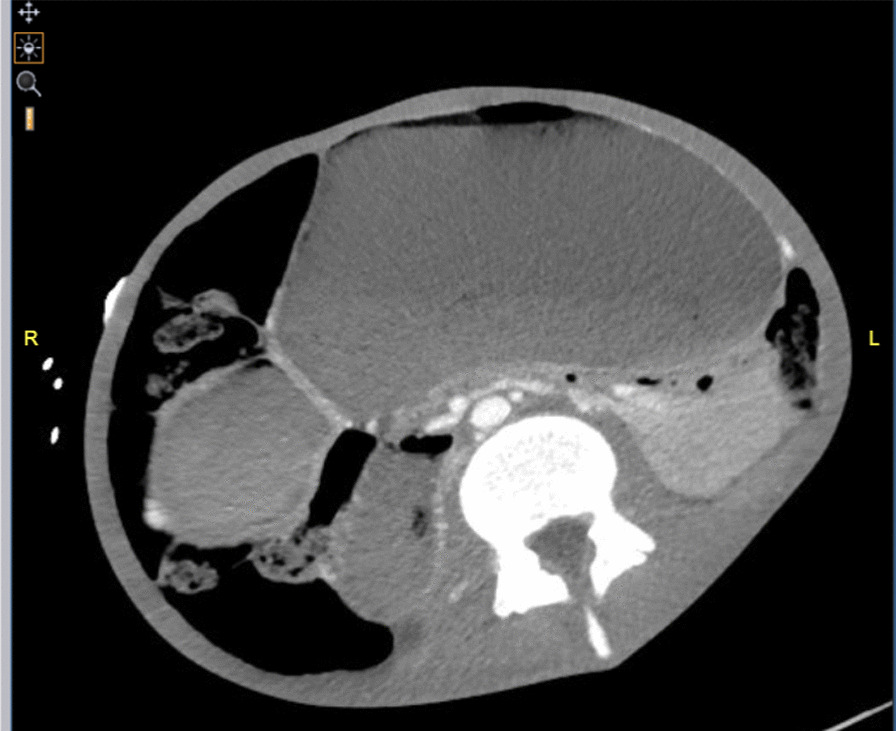
Computed tomography transverse view

The images showed return of normal caliber small bowel within the pelvis, with gas and a very large volume of stool present throughout the colon. The mesentery was compressed posterior to the stomach, and evaluation of small bowel loops was limited by the absolute absence of intraperitoneal fat. There was no free air or free fluid. The hepatobiliary and genitourinary systems were unremarkable (Figs. 2, 3, and 4).

General surgery and gastroenterology were consulted. Obstruction and perforation of the small bowel were ruled out by esophagogastroduodenoscopy; the stomach was noted to be mildly irritated, but the small bowel was widely patent, and the mucosa was normal.

Her gastric distention was thought to be secondary to reduced gastric dysmotility in the setting of anorexia with frequent binge/purge episodes [1, 4]. Superior mesenteric artery (SMA) syndrome with compression of the duodenum between the aorta and SMA was considered but deemed less likely on the differential diagnosis; it is a known, albeit rare, complication in patients with eating disorders presenting with acute gastric distention [7, 8]. No further work up of SMA syndrome took place as the patient improved with conservative therapy. Decompression of the stomach followed by refeeding was commenced through nasogastric tube. Within 2 days, her constipation resolved through treatment with promotility agents and rectal sodium phosphate laxatives.

The medicine team worked with the patient, the psychiatry team, and the dietician to increase her oral intake until she was medically stable for transfer to the inpatient eating disorders program. However, clinical challenges arose related to her worsening anorexia symptoms, which included refusing refeeding and intention to leave hospital, despite not being medically fit. A full interdisciplinary team approach was utilized in order to ensure standard of care for the patient.

## Discussion and conclusions

Acute gastric distention is caused by a myriad of etiologies which may cause ischemic injury to the stomach [6, 7]. These causes include gastroparesis, eating disorders, electrolyte imbalances, psychogenetic polyphagia, and obstructions. The diagnosis therefore needs to be promptly made in order to rule out gastric perforation and hemorrhage. This is of key importance as the sequalae of gastric ischemia and perforation due to acute dilatation have been reported to have a mortality rate of 80% to 100% [8, 9]. Despite the extensive gastric collateral circulation, acute intragastric venous pressures greater than 14 mmHg may lead to impaired intramural blood flow and can therefore cause mucosal necrosis [9]. Dilatations causing volume increases of more than 3 to 5 l from baseline can cause mucosal tears and cause ischemic injury [9].

Clinically, symptoms tend to be non-specific but may include vomiting, nausea, and mild to severe abdominal distention. Rupture may lead to more pronounced symptomatology including peritoneal signs [4]. History of underlying disorders or predisposing disorders is key for clinical suspicion and decision making. The definitive diagnostic test is made by imaging.

Radiographically, abdominal x-ray may reveal a dilated stomach, and in the presence of gastric perforation may show pneumo-peritoneum on erect chest x-ray. A CT scan will show a dilated stomach; furthermore, CT can reveal alternative causes of AGD including superior mesenteric artery syndrome, although arteriogram may be necessary if the diagnosis is unclear. Further diagnostic clarification can be made with upper gastrointestinal endoscopy which can detect gastric outlet obstructions and early signs of ischemia [4, 9]. If dysmotility issues are suspected, gastric scintigraphy is required to establish the diagnosis.

The definitive treatment of AGD largely depends on the etiology and may include surgery for patients with perforation or extensive ischemic injury. First-line acute treatment of AGD consists of nasogastric decompression and fluid resuscitation.

One of the most common mechanistic theories for acute gastric dilation in patients with anorexia is gastroparesis (delayed gastric emptying), which develops frequently due to food restriction with weight loss. Typically, this phenotype presents with bloating postprandially [10–12]. However, more nuanced physiological mechanisms have been proposed for the mechanism of acute gastric dilation in eating disorders. For example, one study found that bulimia nervosa patients demonstrated delayed gastric emptying and diminished gastric relaxation. In addition, diminished release of cholecystokinin and abnormalities in enteric autonomic function were found in bulimia nervosa patients [11]. However, the exact pathogenesis remains unclear.

Our case report adds to the literature of rare occurrences of AGD in patients with anorexia nervosa. It highlights the importance of diagnostic steps in patients who present with a psychiatric condition and a concurrent potentially catastrophic medical situation. More importantly it demonstrates the complexities of the underlying pathophysiology of AGD, as our patient had no obvious obstructive cause of her AGD, and the underlying reasons for anorexia-associated AGD remain unclear.

## Data Availability

Not applicable to this article as no datasets were generated or analyzed.

## Abbreviations

AGD: Acute gastric dilatation
CT: Computed tomography
SMA: Superior mesenteric artery
